# (+)-Usnic Acid as a Promising Candidate for a Safe and Stable Topical Photoprotective Agent

**DOI:** 10.3390/molecules26175224

**Published:** 2021-08-28

**Authors:** Agnieszka Galanty, Justyna Popiół, Magdalena Paczkowska-Walendowska, Elżbieta Studzińska-Sroka, Paweł Paśko, Judyta Cielecka-Piontek, Elżbieta Pękala, Irma Podolak

**Affiliations:** 1 Department of Pharmacognosy, Faculty of Pharmacy, Jagiellonian University Medical College, Medyczna 9, 30-688 Krakow, Poland; irma.podolak@uj.edu.pl; 2 Department of Pharmaceutical Biochemistry, Faculty of Pharmacy, Jagiellonian University Medical College, Medyczna 9, 30-688 Krakow, Poland; justyna.popiol@uj.edu.pl (J.P.); elzbieta.pekala@uj.edu.pl (E.P.); 3 Department of Pharmacognosy, Poznań University of Medical Sciences, Święcickiego 4, 60-781 Poznan, Poland; mpaczkowska@ump.edu.pl (M.P.-W.); ela_studzinska@op.pl (E.S.-S.); jpiontek@ump.edu.pl (J.C.-P.); 4 Department of Food Chemistry and Nutrition, Faculty of Pharmacy, Jagiellonian University Medical College, Medyczna 9, 30-688 Krakow, Poland; p.pasko@uj.edu.pl

**Keywords:** usnic acid, photoprotection, normal skin cells, octocrylene

## Abstract

The study aimed to examine whether usnic acid—a lichen compound with UV-absorbing properties—can be considered as a prospective photoprotective agent in cosmetic products. Moreover, a comparison of two usnic acid enantiomers was performed to preselect the more effective compound. To meet this aim, an in vitro model was created, comprising the determination of skin-penetrating properties via skin-PAMPA assay, safety assessment to normal human skin cells (keratinocytes, melanocytes, fibroblasts), and examination of photostability and photoprotective properties. Both enantiomers revealed comparable good skin-penetrating properties. Left-handed usnic acid was slightly more toxic to keratinocytes (IC_50_ 80.82 and 40.12 µg/mL, after 48 and 72 h, respectively) than its right-handed counterpart. The latter enantiomer, in a cosmetic formulation, was characterized by good photoprotective properties and photostability, comparable to the UV filter octocrylene. Perhaps most interestingly, (+)-usnic acid combined with octocrylene in one formulation revealed enhanced photoprotection and photostability. Thus, the strategy can be considered for the potential use of (+)-usnic acid as a UV filter in cosmetic products. Moreover, the proposed model may be useful for the evaluation of candidates for UV filters.

## 1. Introduction

Usnic acid (2,6-diacetyl-7,9-dihydroxy-8,9b-dimethyl-1,3(2*H*,9b*H*)-dibenzofurandione) is a dibenzofuran derivative, synthesized exclusively by lichens. Over the past several years, this compound has gained much attention due to the broad spectrum of its biological and pharmacological activities, combined with its high availability from natural sources (content up to 10%). Usnic acid is yellow, lipophilic, and chiral, with two enantiomers, of which (+)-usnic acid is more prevalent in lichen species. The question regarding differences in the activity of both enantiomeric forms is still open, as the evidence supporting the advantages of one form over the other is scarce and unambiguous, as recently reviewed in our previous work [1]. Although usnic acid reveals marked cytotoxic, anti-inflammatory, and antibacterial properties [2], some studies pointed to its hepatotoxicity [3,4], which may limit the internal administration of this compound. On the other hand, a few case studies have reported on contact allergy to usnic acid, but extracts with its compounds have been successfully used in antiperspirants [1]. Thus, topical application of usnic acid seems to be a more promising approach as regards its future use.

Several studies show a clear relationship between usnic acid content in lichens and the intensity of solar radiation at their location [5,6,7,8]. This phenomenon may be linked to the UV-absorbing properties of usnic acid, thanks to which it protects lichens from the damaging effects of UV light exposure. The physiological protective role of usnic acid has been further examined in several in vitro experiments on irradiated skin cells [9,10,11]. A single animal study on irradiated guinea pigs also confirmed that usnic acid could be considered as a potential sunscreen agent, with SPF two times higher as compared to the reference (homosalate) [12]. The effectiveness of usnic acid in sun protection was also tested on human volunteers, and the results were comparable to a reference Nivea sun spray (SPF 5) [13]. This feature may be an interesting option in the development of new lichen-based cosmetic products, especially since usnic acid is a well-known ingredient that has been already used by the cosmetics industry in antiperspirants and toothpaste [1]. However, as the number of studies focused on the photoprotective potential of usnic acid is still very limited, further experiments are required in order to also verify the safety of the compound. 

Excessive ultraviolet radiation, which exerts a negative impact on human skin, is emerging as a health problem that is closely connected with a progressing depletion in the ozone layer over our planet [14]. To prevent skin damage, cosmetic products with various UV filters are recommended for regular use. One such well-known UV filter is octocrylene (2-ethylhexyl 2-cyano-3,3-diphenylacrylate), which mainly absorbs UVB radiation. It has been successfully used in cosmetic products as a sunscreen agent or to protect cosmetic formulations from UV radiation [15]. According to recent studies, some of the commercial UV filters may show adverse effects, e.g., an impact on the endocrine system, inducement of skin allergy, or toxicity to some marine organisms [16,17]. Thus, there is a constant need to search for new candidates to be used as sunscreen agents that show good photoprotective activity along with high photostability and safety. One of the possible approaches involves combining two or more UV filters in one formulation to obtain an enhancement of the effect. This could allow the lowering of the doses of single components and, hence, an increase in the safety of such a product, while securing a comparable protective effect. In the case of usnic acid, such an approach has proved to be successful, as was reported in several recently published papers, where its combination with other drugs gave satisfactory results in cytotoxicity studies or against resistant bacteria strains [18,19]. Thus, the current study aimed to examine and compare the skin-penetrating properties and safety profiles of two usnic acid enantiomers toward normal human skin cells in vitro. The preselected enantiomer was then examined for its photoprotective effectiveness and photostability. Moreover, the effect of the preselected usnic acid enantiomer, combined with the UV filter octocrylene, was also studied. 

## 2. Results

### 2.1. Both Usnic Acid Enantiomers Reveal High Skin Permeability

The permeability of usnic acid enantiomers was tested and compared by using the in vitro skin-PAMPA model. The results of the permeability test are presented in Table 1*,* calculated as apparent permeability coefficients (P_app_). The obtained values P_app_ for both (+)- and (−)-usnic acid indicated their high ability to penetrate the skin barrier, but the effect was not dose-dependent. Moreover, no significant differences were observed between the permeability coefficients calculated for both enantiomers (*p* > 0.05).

### 2.2. (+)-Usnic Acid Is Safer to Normal Skin Cells than Its Left-Handed Enantiomer

**Figure 1:**
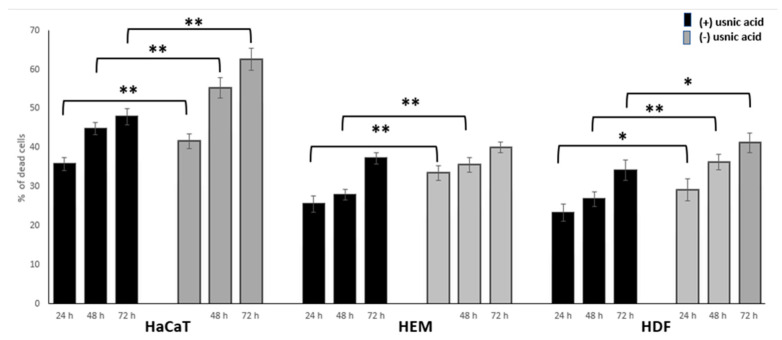
Cytotoxic activity of (+)- and (−)-usnic acid towards normal HaCaT skin keratinocytes, HEM melanocytes and HDF fibroblasts, at the highest tested concentration of 100 µg/mL, after 24, 48, and 72 of incubation (differences statistically significant: * *p* < 0.05; ** *p* < 0.01).

A toxicity study was performed by means of the lactate dehydrogenase (LDH) viability test. Both usnic acid enantiomers revealed high safety to the tested skin cells. Among the skin cells used in our model, HaCaT keratinocytes were the most sensitive (Figure 1), but the overall toxicity of usnic acid enantiomers was low, and observed only at more prolonged exposure and the highest tested concentration, with IC_50_ values for (−)-usnic acid of 80.82 and 70.12 µg/mL after 48 and 72 h, respectively, and with IC_50_ > 100 µg/mL for the remaining cell lines. Statistically significant differences in the impact of both enantiomers (Figure 1) on cell viability were observed, with slightly higher toxicity of the left-handed enantiomer (*p* < 0.01 for HaCaT after 24, 48, 72 h of exposure, HEM cells after 24 and 48 h of exposure, and HDF cells after 48 h of exposure; *p* < 0.05 for HDF cells after 24 and 72 h of exposure).

### 2.3. (+)-Usnic Acid Absorbs UV Radiation Similarly to Octocrylene

**Figure 2:**
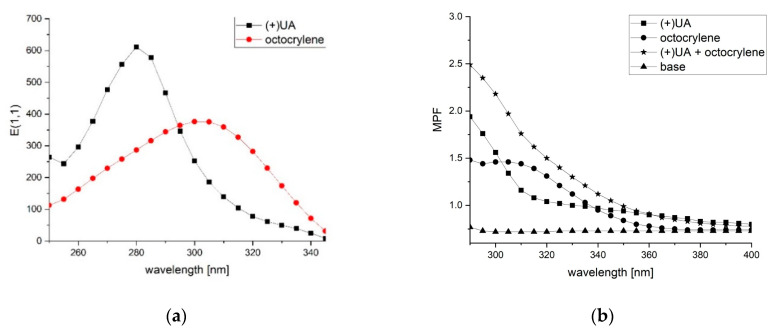
UV absorption spectra of: (**a**) (+)-usnic acid (+UA) and the reference UV filter octocrylene, obtained in ethanol solutions.; (**b**) (+)-usnic acid (+UA), octocrylene, and (+)-usnic acid with octocrylene, obtained in a cosmetic formulation at 1% (*w/w*) concentration applied on polymethylmethacrylate plates (MPF: monochromatic protection factor).

Ultraviolet spectroscopic properties of (+)-usnic acid and the reference UV filter octocrylene, in ethanol solutions, were measured by recording their absorption spectra between 250 and 400 nm, and the results are presented in Table 2 and Figure 2a. Right-handed usnic acid absorbed ultraviolet radiation in a similar range to octocrylene (from 250 to 347 nm), and showed a significantly higher molar extinction coefficient (Table 2). Despite this, (+)-usnic acid revealed more favorable spectroscopic properties in the UVC region, which is well illustrated in Figure 2a. The λ_max_ of (+)-usnic acid is located at 281 nm, but this subtype of ultraviolet radiation is absorbed by the ozone layer, and is not considered when new UV filters are developed. Analyzing the absorption profiles of (+)-usnic acid and octocrylene in the UVB and UVA regions, it can be seen that at 290 nm the extinction at 1 cm path length and 1% concentration (E1,1) value is higher for the former compound, at 295 nm the E1,1 value is similar for both compounds and, finally, at 300 nm octocrylene achieves a stronger absorption profile than (+)-usnic acid (Figure 2a).

### 2.4. (+)-Usnic Acid Enahances the Photoprotective Potential of Octocrylene

The photoprotective activity of the tested compounds in a cosmetic formulation was determined by the diffuse transmittance; results are presented in Table 3 and Figure 2b. All of the treatment conditions differed significantly from the cosmetic base, used for control conditions (*p* < 0.001). (+)-Usnic acid and octocrylene showed comparable activity in the UVB region, with slightly (~12%) higher SPF_in vitro_ for the latter (*p* < 0.01). After adding (+)-usnic acid to the formulation containing octocrylene, SPF_in vitro_ and UVA PF increased by 25 and 13.25%, respectively, when compared to octocrylene alone (*p* < 0.001), suggesting the enhanced effect of both compounds.

### 2.5. Increase in Photostability of the Formulation with (+)-Usnic Acid Combined with Octocrylene 

**Figure 3:**
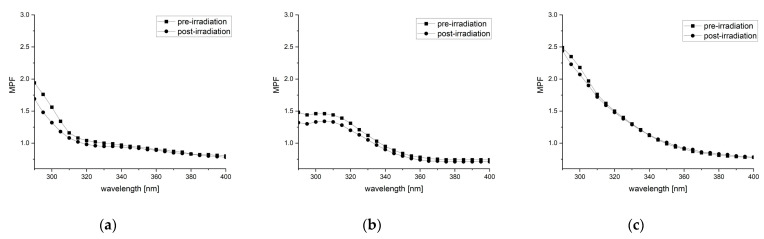
UV absorption spectra of the tested formulations both pre-irradiation and after 1 h of irradiation with a solar light simulator at 500 W/m^2^: (**a**) 1% (+)-usnic acid; (**b**) 1% octocrylene; (**c**) (+)-usnic acid with octocrylene, obtained in a cosmetic formulation at 1% (*w*/*w*) concentration (MPF: monochromatic protection factor).

Changes in the absorption in the UVA and UVB regions of formulations containing (+)-usnic acid, octocrylene, or both compounds, followed by irradiation with a solar light simulator at 500 W/m^2^ (cumulative dose of ultraviolet radiation 218 kJ/m^2^), were examined. The results, presented in Table 4 and Figure 3, indicated that after irradiation, SPF_in vitro_ values for the formulations containing (+)-usnic acid and octocrylene decreased by 8.37 and 7.38%, respectively. The most important results were observed for the formulation containing both (+)-usnic acid and octocrylene, where the decrease in SPF_in vitro_ was only 2.35%, and the result differed significantly from those obtained for each compound used alone (*p* < 0.001), indicating the high photostability of the mixture. 

## 3. Discussion

A candidate compound for topical use should be verified for its potential to penetrate the skin barrier, as well as its safety profile, together with photoprotective potential and photostability. This study focused on the preselection of a novel candidate for a UV filter from among the two usnic acid enantiomers, considering whether the compounds met the above-mentioned criteria. Moreover, the preselected usnic acid enantiomer was combined with a known UV filter (octocrylene) in a cosmetic formulation, in order to reveal the potential enhancement. To achieve the assumed aim, an in vitro model was then created, consisting of the skin-PAMPA assay, LDH viability test on normal skin cells, and a combination of spectrophotometric analyses, in order to determine skin-penetrating properties, safety, photostability, and photoprotective properties. Such an approach provides quick verification of the properties of the examined compounds, measured under the same laboratory conditions, thus enabling the preselection of the candidate for further studies. 

In the first step of our study, the permeability of both usnic acid enantiomers was measured and compared using the in vitro skin-PAMPA assay, based on passive diffusion, followed by the determination of their toxicity towards skin cells. Both (+)- and (−)-usnic acid revealed their high ability to penetrate the skin barrier, but no significant differences were observed between the two enantiomers (*p* > 0.05). The high penetrating potential of usnic acid, likely resulting from its lipophilic properties [20], was determined for the first time with the use of a skin-PAMPA test. Previously, some other in vitro permeability tests were used to determine the penetrating potential of usnic acid—namely, Franz cells and porcine skin, combined with HPLC quantitative analysis [21,22]—but the whole experiment lasted longer. Thus, the skin-PAMPA assay, combined with spectrophotometric quantitative analysis, might be a time- and cost-saving alternative model for the preliminary assessment of skin-penetrating potential.

In order to determine the safety profiles of both usnic acid enantiomers towards the skin, we performed cytotoxicity experiments on a panel of normal skin cells, representing different skin layers—namely, keratinocytes (HaCaT), melanocytes (HEM), and fibroblasts (HDF). Cell viability was tested in a wide concentration range (5–100 µg/mL), not only after the standard 24 h of incubation, but also after longer exposure (48 and 72 h), in order to determine the in vitro biokinetics of the tested compounds. Both usnic acid enantiomers revealed high safety towards the tested skin cells; however, (+)-usnic acid was less toxic than its left-handed enantiomer (*p* < 0.001). HaCaT keratinocytes were the most sensitive to the tested compounds, as was observed especially with the higher doses used during the experiment. Keratinocytes, forming the outermost layer of the human skin, are the first barrier against the damage caused by environmental factors, including excessive UV radiation. At the same time, their ability to proliferate and revive is higher than that of the cells from deeper skin layers. Thus, the observed toxic effects after long exposure to high doses of usnic acid enantiomers, similar to those appearing in the organism, may be acceptable. Our results for HaCaT cells are consistent with those described by [23] for usnic acid, with an IC_50_ of 185.7 ± 4.8 µM (about 64 µg/mL). Similarly low toxicity of (+)-usnic acid towards fibroblasts was proven in our previous study, with an IC_50_ > 40 µg/mL [24], and by [25] for HDF cells, with an IC_50_ > 10 µM (about 3.5 µg/mL). Interestingly, the only published study on the impact of usnic acid on HEM cells revealed its high toxicity, with an IC_50_ of 6.9 µM (about 2.4 µg/mL) [26], while our results suggest the opposite; thus, this problem requires further study. Our in vitro model, representing different skin layers, may mimic the complex nature of the skin itself. It can be used for obtaining thorough information on the impact of usnic acid on skin cells of different origin, as an alternative to animal studies. Moreover, our study is likely the first to determine and compare the toxicity of both usnic acid enantiomers towards normal skin cells. 

Right-handed usnic acid was chosen for further evaluation of photoprotective potential, as it is safer towards normal skin cells. We started the evaluation of (+)-usnic acid’s suitability as a prospective UV filter with the determination of its UV absorption profile, compared to the reference UV filter octocrylene. The absorption profiles of both (+)-usnic acid and octocrylene in the UVB and UVA regions were comparable, with the slight predomination of the latter compound at 300 nm. However, it is worth mentioning that, in the cosmetics industry, even weaker UV absorbers are used as UV filters, e.g., dimethicodiethylbenzalmalonate (INCI: Polysilicone-15), with an E1,1 value of ~150 [27]. Based on a comparison of the extinction at 1 cm path length and 1% concentration (E1,1) mean (a mean value of the specific extinction over the spectral range from 290 to 400 nm), (+)-usnic acid may be characterized as only a slightly weaker UV filter than octocrylene. 

The parameters of photoprotective activity, such as SPF and UVA PF, depend not only on the structure and concentration of the compound, but also on the solvent used [28]. The assessment of the photoprotective activity of compounds using a spectrophotometric assay of dilute solutions is considered to be most valuable at an early stage of the development of new sunscreen agents. Hence, to more accurately assess the potential activity of (+)-usnic acid as a UV filter, it was tested in a cosmetic formulation. Moreover, we wanted to examine whether (+)-usnic acid may enhance the photoprotective potential of octocrylene when combined in the same formulation. The photoprotective activity of the tested cosmetic formulations was determined via diffuse transmittance—the in vitro technique recommended to determine some of the photoprotective parameters of cosmetic products [29]. PMMA plates with rough surfaces were chosen as a substrate to imitate the physical characteristics of the skin. Formulations containing 1% (+)-usnic acid, 1% octocrylene, and 1% (+)-usnic acid +1% octocrylene were prepared and tested, and compared to the cosmetic base alone (control). The 1% concentration of (+)-usnic acid was chosen empirically in the preliminary step of the experiment. Perhaps most interestingly, the best results in terms of photoprotective parameters were obtained for the formulation containing both compounds, with a 25 and 13.25% increase in SPF_in vitro_ and UVA PF, respectively, when compared to octocrylene alone. A similar but slightly higher SPF value for usnic acid was described by [30], while UVA PF was almost two times higher than in our experiment. These differences may result from the higher amount of usnic acid (up to 10%) in the formulation tested by the authors, despite using the same in vitro test. In a further study by the same authors, the reported photoprotective parameters of usnic acid were much higher (SPF 3.9, PF UVA 1.8) when compared to our results, and most likely result from the use of a different test for the evaluation [31]. 

The damaging effect of ultraviolet radiation on usnic acid in organic solvents such as methanol is well documented. It was demonstrated that after a three-week exposure of a methanol solution of UA to light (12 h/day natural sunlight +12 h/day in the incubator with a light source), the compound was wholly degraded [32]. Moreover, for some UV filters—such as octinoxate (INCI: ethylhexyl methoxycinnamate, OMC)—the influence of the molecular environment on their photostability has been proven [32]. Thus, we decided to verify the photostability of (+)-usnic acid in a formulation in which it can be potentially used in the future as a UV filter. Hence, in the next step of our experiment, we investigated changes in the absorption in the UVA and UVB regions of formulations containing (+)-usnic acid, octocrylene, or both compounds combined, followed by irradiation with a solar light simulator. As octocrylene is considered to be a photostable UV filter [33], the comparable results obtained for (+)-usnic acid is satisfactory. Moreover, the photostability of (+)-usnic acid is higher in comparison with OMC [13,34]. The results obtained for the formulation containing the combination of (+)-usnic acid and octocrylene are of great importance, as the observed decrease in SPF_in vitro_ was only 2.35%, which indicates the high photostability of the mixture. This is likely the first attempt to combine usnic acid with other commercially used UV filters, and the observed enhancement in photoprotection and higher photostability of the formulation offer a prospective new option for further utilization of usnic acid in the cosmetics industry. 

## 4. Materials and Methods

### 4.1. Materials and Chemicals

Octocrylene was purchased from Sigma-Aldrich, and ethanol absolute for analysis was obtained from Merck. Tween^®^ 60, Lanette^®^ O, Cetiol^®^ CC, Captex^®^ 335 and Cosmedia^®^ Guar were purchased from BASF. Tegosoft^®^ TN was obtained from Evonik Industries, whereas paraffinum liquidum and glycerol were provided by Chempur (Piekary Śląskie, Poland). The concentrated PRISMA HTTM solution, hydration solution, and skin-PAMPA plates were obtained from Pion Inc. (Billerica, MA, USA). Cell culture media (high-glucose DMEM, melanocyte growth medium, fetal bovine serum, trypsin–EDTA solution, and penicillin–streptomycin solution), Triton X100, and DMSO were purchased from Sigma-Aldrich (Seelze, Germany). Human epidermal melanocytes (HEM) and dermal fibroblasts (HDF) were purchased from Sigma-Aldrich, while HaCaT human skin keratinocytes were kindly provided by Prof. Marta Michalik from the Department of Cell Biology, Jagiellonian University in Kraków, Poland. Both (+)- and (−)-usnic acid were isolated, from *Cladonia arbuscula* and *C. uncialis*, respectively, as described previously [4]. Lactate dehydrogenase (LDH) assay kits were obtained from Clontech (Mountain View, CA, USA). 

### 4.2. Permeability Study 

The skin-PAMPA (parallel artificial membrane permeability assay) method was used, as described previously [35], to determine the permeability of the tested usnic acid enantiomers. The model consists of a two-chamber PAMPA sandwich, with 96 wells each. Before the experiment, the top plate, with the lipid-impregnated skin-mimetic membrane, was hydrated overnight with the hydration solution (200 µL per well). The tested substances were added at concentrations of 2 and 4 µg/mL. Each experiment was repeated at least three times, using six replicates on each plate, and the results were presented as mean ± SD. The amount of permeated active compounds was determined by spectroscopic analysis (Multiskan GO microplate reader, Thermo Scientific MaxQ 4450, Waltham, MA, USA), with a wavelength of 290 nm. For the calibration curve, stock solutions in the range of 12.5–150 µg/mL were prepared. 

The apparent permeability coefficients (*P_app_*) were calculated using the following equation:

(1) $$P_{app}=\frac{-ln\left(1-\frac{C_{A}}{C_{equilibrium}}\right)}{S\times \left(\frac{1}{V_{D}}+\frac{1}{V_{A}}\right)\times t}$$

where *V*_D_ = donor volume, *V*_A_ = acceptor volume, *C_equilibrium_* = equilibrium concentration, $C_{equilibrium}=\frac{C_{D}\times V_{D}+C_{A}\times V_{A}}{V_{D}+V_{A}}$, *C_D_* = donor concentration, *C_A_* = acceptor concentration, *S* = membrane area, and *t* = incubation time (in seconds). Compounds with *P_app_* < 1 × 10^−6^ cm/s are classified as having low permeability, and those with *P_app_* > 1 × 10^−6^ cm/s as highly permeable compounds.

### 4.3. Cell Viability Study

Normal human skin keratinocytes (HaCaT), melanocytes (HEM), and fibroblasts (HDF) were grown at 37 °C in a 5% CO_2_ atmosphere, with relative humidity, on high-glucose DMEM (HaCaT, HDF), or melanocyte growth medium (HEM), supplemented with 10% fetal bovine serum (FBS) and antibiotics. Before the experiment, cells were seeded onto 96-well plates (1.5 × 10^4^ cells/well) for 24 h. Then, the culture medium was replaced with the same medium, containing 2–100 µg/mL of the tested substances, and incubated for 24, 48, or 72 h. LDH release was measured as described previously [36]. Briefly, a proper quantity of a reagent mixture was added to the cell supernatants, and after 30 min the absorbance was measured at 490 nm (the reference wavelength 600 nm) with a BioTek Synergy microplate reader (BioTek Instruments Inc., Winooski, VT, USA). Cytotoxicity was calculated as follows: ((A_sample_ − A_control_)/(A_ma_ − A_control_)) × 100; where A_sample_ = the absorbance value for the cells incubated with the tested substances., A_control_ = the absorbance value for untreated, control cells (spontaneous LDH release), and A_max_ = the absorbance value in Triton-X100-lysed cells (maximum LDH release).

Each experiment was performed in triplicate, and the results were presented as mean ± SD.

### 4.4. Ultraviolet Spectroscopy

Spectra with a scan range of 250–400 nm were recorded in 30 µM ethanol solutions, in 1 cm path length, with 1.5 mL quartz cuvettes on a U-2800 double-beam spectrophotometer (Hitachi, Tokyo, Japan) controlled using UV Solution version 2.2 software. The molar extinction coefficient at maximum absorption (ε_max_) of the tested compounds was determined in ethanol as the slope of the linear regression of absorbance vs. the concentration of the tested compound (from 10 to 50 µM). The *E*_1,1_ coefficient was calculated using the formula:

$$E1,1=\varepsilon \left[\frac{L}{mol\;cm}\right]\times \;\frac{10\left[\frac{g}{L}\right]}{M\frac{g}{mol}]}\;\times \;1\left[cm\right]$$

where ‹*E*_1,1_›_mean_ value is a mean value of the specific extinction over the spectral range from 290 to 400 nm. 

### 4.5. Cosmetic Formulations

(+)-Usnic acid and octocrylene, used as a reference standard, were incorporated within a neutral cosmetic formulation at a concentration of 1%. The cosmetic emulsion consisted of Tegosoft^®^ TN, Tween^®^ 60, Lanette^®^ O, Cetiol^®^ CC, paraffinum liquidum, Captex^®^ 335, Glycerol, Cosmedia^®^ Guar, and water. Prior to their incorporation into emulsion, the compounds were mixed with Tegosoft^®^ TN, and then the mixture was heated at 70 °C until dissolved. 

### 4.6. In Vitro Photoprotection Study

An in vitro photoprotection study was performed according to EN ISO 24443:2012 [37], with slight modifications. An accurately weighed formulation with the tested compounds (1.3 mg/cm^2^) was spread across the entire surface of a polymethylmethacrylate plate (PMMA, Schonberg GmbH, Hamburg, Germany), with an application area of 25 cm^2^ and a 5 µm roughness value to simulate the surface of human skin. For each sample, two plates were prepared, and absorbance measures were performed on 6 different positions of the plate from 290 to 400 nm with 1 nm steps. Measurements were carried out via reflectance spectrophotometry with an SPF-290AS Analyzer (Solar Light Company, Glenside, PA, USA) equipped with an integrating sphere controlled by WinSPF version 4.4 software. The results were expressed as the mean from 12 scans. The SPF_in vitro_ and UVA protection factor of the tested formulations were calculated according the following equations:

(2) $$SPF\;in\;vitro=\;\frac{\int_{290}^{400}E\left(\lambda \right)\;I\left(\lambda \right)\;d\lambda }{\int_{290}^{400}E\left(\lambda \right)\;I\left(\lambda \right)\;T\left(\lambda \right)\;d\lambda }\;$$

where *E(λ)* is the erythema action spectrum [23], *I(λ)* is the spectral irradiance received from the UV source (SSR for SPF testing) [22], *T(λ)* is the measured transmittance of the test formulation layer, and *dλ* is the wavelength step (1 nm).

(3) $$UVA\;PF=\;\frac{\int_{320}^{400}P\left(\lambda \right)\;I\left(\lambda \right)\;d\lambda }{\int_{320}^{400}P\left(\lambda \right)\;I\left(\lambda \right)\;T\left(\lambda \right)\;d\lambda }\;$$

where *P(λ)* is the PPD action spectrum [37], *I(λ)* is the spectral irradiance received from the UV source (SSR for SPF testing) [37], *T(λ)* is the measured transmittance of the test formulation layer, and *dλ* is the wavelength step (1 nm). 

### 4.7. Photostability Study

To assess the functional photostability of formulations containing the tested compounds, the PMMA plates with tested samples were irradiated using a solar light simulator (SUNTEST CPS+, Atlas, Linsengericht, Germany) equipped with an optical filter cutting off wavelengths shorter than 290 nm and an IR-block filter to neutralize thermal effects. Samples were irradiated at 500 W/m^2^ for 1 h (cumulative dose of ultraviolet radiation 218 kJ/m^2^). The solar simulator’s emission and irradiation time were in accordance with previous studies on the photodegradation of sunscreen agents [38,39]. The UV absorption spectra and photoprotective activity parameters (SPF_in vitro_, *UVA PF*) of the samples were analyzed post-irradiation and compared with pre-irradiation results. 

### 4.8. Statistical Analysis

The differences between usnic acid enantiomers were tested using one-way analysis of variance (ANOVA) with the Tukey–Kramer multiple comparisons post hoc test. Statistical analysis was done using Statistica v.13 (StatSoft, Tulsa, OK, USA).

## 5. Conclusions

Right-handed usnic acid turned out to be a good candidate for a UV filter for topical use, in terms of its ability to penetrate the skin barrier, safety to skin cells, photoprotection, and photostability. The availability of the compound is also good, as it is present in high amounts in lichen species (e.g., *Usnea*, *Cladonia*, *Ramalina* spp.), from which it may be easily obtained. It can be also synthesized in order to avoid overexploitation of its natural sources. The combination of (+)-usnic acid with octocrylene in one formulation not only gave the most promising results in terms of photoprotection, but also significantly improved the photostability of the mixture, which is an interesting strategy to be followed in future studies. The incorporation of (+)-usnic acid in the sunscreen formulation may reduce the concentration of UV filters in the final product and improve their efficacy and safety. 

## Figures and Tables

**Table 1:** Apparent permeability values (P_app_) for (+)- and (−)- usnic acid.

Concentration (µg/mL)	P_app_ ± SD (×10^−6^ cm s^−1^)	
	(+)-Usnic Acid	(−)-Usnic Acid
2	7.53 ± 1.25	7.46 ± 1.12
4	7.90 ± 0.65	8.28 ± 1.33

**Table 2:** Ultraviolet spectroscopic properties of (+)-usnic acid and the reference UV filter octocrylene, obtained in ethanol solutions.

Compound	λ_max_ (nm)	ε_max_ (M^−1^ cm^−1^)	E1.1 (λ_max_)	*‹*E_1.1mean_*›*
(+)-Usnic acid	281	21 120	613	76
Octocrylene	302	13 650	378	141

**Table 3:** Photoprotective activity (SPF_in vitro_, UVA PF) of the tested formulations.

Formulation	SPF_in vitro_ ± SD	UVA PF ± SD
1% (+)-usnic acid	1.20 ± 0.08 ^#,a,b^	0.86 ± 0.04 ^#,d,e^
1% octocrylene	1.36 ± 0.02 ^#,a,c^	0.83 ± 0.01 ^#,d,f^
1% (+)-usnic acid + 1% octocrylene	1.70 ± 0.07 ^#,b,c^	0.94 ± 0.01 ^#,e,f^
Control (base)	0.72 ± 0.01	0.70 ± 0.01

^#^ Differences statistically significant from the control (*p* < 0.001); the same letters in superscript indicate statistically significant differences between the individual data within each column (^a^: *p* < 0.01; ^b, c, e, f^: *p* < 0.001, ^d^: *p* < 0.05).

**Table 4:** The changes in the photoprotective activity of the tested formulations after irradiation with a solar light simulator at 500 W/m^2^.

Formulation	% of Initial SPF_in vitro_ ± SD	% of Initial UVA PF ± SD
1% (+)-usnic acid	91.63 ± 0.59 ^a^	97.67 ± 0.00 ^c^
1% octocrylene	92.62 ±1.57 ^b^	95.15 ± 0.86 ^d^
1% (+)-usnic acid + 1% octocrylene	97.65 ± 5.82 ^a,b^	101.06 ± 3.01 ^c,d^

The same letters in superscript indicate statistically significant differences between the individual data within each column (*p* < 0.001).

## Data Availability

Data available on request.

## Sample Availability

Samples of the compounds (usnic acid enenatiomers) are available from the authors.
